# Research on Volatile Organic Compounds From *Bacillus subtilis* CF-3: Biocontrol Effects on Fruit Fungal Pathogens and Dynamic Changes During Fermentation

**DOI:** 10.3389/fmicb.2018.00456

**Published:** 2018-03-14

**Authors:** Haiyan Gao, Peizhong Li, Xinxing Xu, Qing Zeng, Wenqiang Guan

**Affiliations:** ^1^School of Life Sciences, Shanghai University, Shanghai, China; ^2^Shanghai Key Laboratory of Bio-Energy Crops, Shanghai, China; ^3^Tianjin Key Laboratory of Food Biotechnology, College of Biotechnology and Food Science, Tianjin University of Commerce, Tianjin, China

**Keywords:** *Bacillus subtilis*, volatile organic compounds (VOCs), biocontrol, fruit fungal pathogens, *Monilinia fructicola*, *Colletotrichum gloeosporioides*

## Abstract

The dynamic changes of the levels of volatile organic compounds (VOCs) produced by *Bacillus subtilis* CF-3 and their biocontrol effects on common fungal pathogens were researched in this study. The results showed that the VOCs in 24-h fermentation liquid (24hFL) of *B. subtilis* CF-3 inhibited mycelial growth of *Botrytis cinerea, Colletotrichum gloeosporioides, Penicillium expansum, Monilinia fructicola*, and *Alternaria alternata*, with a mean inhibition rate of 59.97%. The inhibitory effect on *M. fructicola* and *C. gloeosporioides* was the highest; they were therefore selected as target fungal pathogens for further experiments. Based on headspace solid-phase microextraction combined with gas chromatography-mass spectrometry (HS-SPME-GC-MS), 74 potential VOCs were identified during the fermentation: 15 alcohols, 18 ketones, 4 pyrazines, 4 esters, 10 acids, 5 phenols, 3 hydrocarbons, 3 amines, 2 aldehydes, 5 ethers, and 5 other components. At different fermentation times, the type and content of VOCs were different. Most of the potential VOCs (62 VOCs) were identified in the 48hFL. The inhibition rates of all VOCs reached their peaks (73.46% on *M. fructicola* and 63.63% on *C. gloeosporioides*) in the 24hFL. Among the identified VOCs, 2,4-di-*tert*-butylphenol, 1-octanol, and benzothiazole showed significant positive correlations with the rates of *M. fructicola* and *C. gloeosporioides* inhibition. Benzoic acid and benzaldehyde showed a significant positive correlation with the rates of *M. fructicola* inhibition, and anisole and 3-methylbutanal showed a significant positive correlation with the rates of *C. gloeosporioides* inhibition. *In vitro*, 2,4-di-*tert*-butylphenol showed a strong inhibitory effect on both *M. fructicola* and *C. gloeosporioides*. *In vivo*, benzothiazole showed the strongest inhibitory effect on the mycelial extensions of both *M. fructicola* and *C. gloeosporioides*, which also led to an increased rate of healthy fruit. The results of the present study clarified that 2,4-di-*ter*t-butylthiophenol and benzothiazole are key inhibitory VOCs produced by *B. subtilis* CF-3.

## Introduction

Postharvest decay caused by fungal pathogens is one of the main factors limiting the storage life of fruit, and it results in substantial economic losses, especially in the fruit market supply chain (Prusky, 2011). The control of postharvest fruit diseases formerly depended on cold or controlled atmosphere storage and fungicides (Zheng et al., 2013). Fungicides are widely used to limit postharvest decay and extend the shelf-life of fruit. However, they are becoming less effective, owing to the development of pathogen resistance, which results in the overuse and misuse of fungicides and causes a public health risk (Schreinemachers and Tipraqsa, 2012). Despite these problems, fungicides are currently essential for ensuring that the food supply meets the demands of the increasing global population (Wang and Liu, 2007). Therefore, alternative methods are needed to address the concerns related to global environmental contamination and human health risks caused by fungicide residues.

The use of biocontrol microorganisms is a promising strategy for the control of postharvest diseases (Zheng et al., 2013). Many antagonistic microorganisms, e.g., *Aureobasidium pullulans* (Mari et al., 2012), *Metschnikowia fructicola* (Banani et al., 2015), and *Bacillus amyloliquefaciens* (Arrebola et al., 2010), exhibit biocontrol activity that can control the growth of postharvest fungal pathogens on fruit. Among these common biocontrol microorganisms, *Bacillus subtilis* is widely used in agricultural biocontrol applications and to promote plant growth (Ma et al., 2015). Previous studies have indicated that *B. subtilis* can inhibit the mycelial growth of many fungal pathogens (Chen et al., 2008; Leelasuphakul et al., 2008), including *Penicillium expansum* (Senthil et al., 2011), *Monilinia fructicola* (Casals et al., 2012), and *Botrytis cinerea* (Maachia et al., 2015). *B. subtilis* produces a number of antifungal compounds that have important functions in biocontrol, such as lipopeptides (e.g., surfactin, iturin, and fengycin) (Torres et al., 2016) and antifungal enzymes [e.g., chitinase (Liu et al., 2011) and chitosanase (Wang and Yeh, 2008)].

However, while most previous studies have focused on the antifungal proteins of *B. subtilis*, little is known about its volatile organic compounds (VOCs). Recently, VOCs produced by *B. subtilis* have been proposed as an alternative control method for postharvest fruit diseases (Zheng et al., 2013) because they can inhibit the mycelial growth and spore germination of various pathogenic fungi (Chen et al., 2008). For instance, various VOCs (e.g., 2-nonanone, 2-methylpyrazine, and β-benzeneethanamine) produced by *B. subtilis* TB09 and TB72 effectively controlled the anthracnose pathogen on postharvest mangoes (Zheng et al., 2013), and VOCs produced by *B. subtilis* PPCB001 inhibited growth of *Penicillium crustosum* by over 50%, reducing postharvest decay in citrus fruits (Arrebola et al., 2010).

Research into the VOCs produced by *B. subtilis* has thus far primarily focused on identifying exact VOC components and evaluating their biocontrol effects. However, research into dynamic changes in VOCs during microbial fermentation and their biocontrol effects is still scarce. Because the activity and metabolic capacity of a biocontrol strain, as well as the types of VOCs secreted, may change during fermentation (Wang et al., 2016), it is necessary to study the dynamic change in VOCs and biocontrol activity during fermentation in order to determine which compounds play the most important roles in pathogen inhibition.

In a previous study, we isolated, identified, and named the *B. subtilis* strain CF-3, which exhibited biocontrol effects (Gao et al., 2016). Moreover, the VOCs produced by *B. subtilis* CF-3 were also identified after 48 h of cultivation (Gao H. Y. et al., 2017). Considering these findings, the objectives of the present study were: (1) to evaluate the antifungal spectrum and inhibitory effects of VOCs produced by *B. subtilis* CF-3 on common fungal pathogens *in vitro*; (2) to identify the dynamic changes in the levels of VOCs secreted by *B. subtilis* CF-3 during fermentation using headspace solid-phase micro-extraction combined with gas chromatography-mass spectrometry (HS-SPME-GC-MS); (3) to discover the correlation between compounds and inhibition rates by evaluating the dynamic inhibition rates of VOCs secreted from *B. subtilis* CF-3; and (4) to ascertain which compounds were key factors in the inhibition of fungal pathogens both *in vivo* and *in vitro*.

## Materials and methods

### *Bacillus* strain

*Bacillus subtilis* CF-3 (registered in the China Center for Type Culture Collection, CCTCC M 2016125) was isolated from fermented bean curd and identified by the Laboratory of Food Safety and Quality Control (School of Life Sciences, Shanghai University) (Gao et al., 2016). For short-term storage, *B. subtilis* CF-3 was incubated at 37°C for 24 h on Luria-Bertani (LB) agar (Gao H. Y. et al., 2017). All chemicals used were purchased from Sinopharm Chemical Reagent Co., Ltd. (Shanghai, China).

### Pathogenic fungi

The following common fungal pathogens of fruit were selected for this study: *Botrytis cinerea*, responsible for gray mold on strawberries and tomatoes (Wang et al., 2013; Ugolini et al., 2014); *Colletotrichum gloeosporioides*, which causes anthracnose in litchis (Sivakumar et al., 2008); *Penicillium expansum*, responsible for blue mold on apples (Spadoni et al., 2015); *Monilinia fructicola*, which causes peach brown rot (Zhang et al., 2015); *Alternaria alternata*, which causes Alternaria rot and black spot on jujube (Yan et al., 2015); and *Macrophoma kuwatsukai*, which promotes ring rot in apples (Xu et al., 2015). *A. alternata, B. cinerea, M. kuwatsukai*, and *P. expansum* were provided by the College of Horticulture and Landscape, Tianjin Agricultural University; *C. gloeosporioides* was provided by the Chinese Academy of Tropical Agricultural Sciences Environment and Plant Protection Institute; and *M. fructicola* was provided by the Laboratory of Agricultural Products Processing and Storage, Food Science and Engineering College, Beijing University of Agriculture. They were plated onto potato dextrose agar (PDA) medium (Gao H. Y. et al., 2017) in Petri dishes (Ø90 mm) and incubated at 28°C for 7 days prior to use.

### Preparation of *B. subtilis* CF-3 fractions

After 24 h of incubation on LB agar, *B. subtilis* CF-3 was gently scraped and transferred into 100 mL of LB liquid medium in a conical flask and cultivated at 37°C in a rotary shaker at 150 rpm for 24 h. Subsequently, approximately 1 × 10^8^ colony forming units (CFU)/mL were counted using a hemocytometer. Afterwards, 4.15 mL of the prepared *B. subtilis* CF-3 liquid was transferred into 41.5 mL of fresh culture solution, which was then sealed with a silicone pad and aluminum cap in a custom-made conical flask with side arms bound with eight layers of sterile gauze (Gao H. Y. et al., 2017). Eight such conical flasks were prepared and placed in a 150-rpm rotary shaker at 37°C for 12–96 h: the flasks were taken out one by one at 12-h intervals, i.e., at 12, 24, and 36 h, and so forth. The fermentation liquid obtained after shaking the flask cultivated for 12 h was termed the 12-h cultivated fermentation liquid (12hFL), and the remaining fermentation liquids were named in the same manner (24hFL, 36hFL, and so forth). The cell-free filtrate (CFF) of *B. subtilis* CF-3 from the 24hFL was obtained using the method reported by Jiang et al. (2014). A bacterial suspension (BS) of *B. subtilis* CF-3 was obtained from by diluting the bacterial pellet obtained by centrifugation of the 24hFL with tryptone-buffered saline (TPS; 1 g tryptone and 8.5 g NaCl, diluted with distilled sterile water to 1 L, pH = 7.0), and the cell concentration was adjusted to the approximate cell concentration of the 24hFL. The CFF and BS were selected to explore whether the bacterium or its secretions have a stronger inhibitory effect on the fungi.

### Fruit preparation

Peaches (*Prunus persica* cv. DaJiubao) and litchi (*Litchi chinensis* Sonn.) were harvested in orchards in the Shandong and Guangxi provinces, China, respectively. After being harvested and transported to the laboratory, the fruits were processed immediately. Fruits were selected based on size uniformity, ripeness, and the absence of visual injuries or infections. Subsequently, they were superficially disinfected by immersion in 0.1% (v/v) sodium hypochlorite for 1 min, rinsed with tap water, and air-dried at room temperature (25°C).

### Preparation of spore suspensions of pathogenic fungi

Pathogenic fungi were prepared as spore suspensions by flushing the surface of the PDA medium containing Tween 80 diluted in distilled water to 0.05% (v/v) that was used for cultivating the fungi for 7 days at 28°C. The liquid was then filtered through eight layers of sterile cheesecloth, and the concentration was adjusted to 1 × 10^5^ conidia/mL using distilled water.

### Inhibitory effects of VOCs produced by *B. subtilis* CF-3 on pathogenic fungi *in vitro*

The 24hFL, CFF of the 24hFL, and BS of the 24hFL of *B. subtilis* CF-3 were used as three treatments, and *A. alternata, B. cinerea, M. kuwatsukai, P. expansum, C. gloeosporioides*, and *M. fructicola* were selected as the target pathogenic fungi for the experiments. The inhibition rate was evaluated by mycelial growth of the fungi. The inhibitory effects of VOCs produced by fermentation and the components of the fermentation liquids of *B. subtilis* CF-3 were evaluated using the methods described by Arrebola et al. (2010). In brief, LB agar plates (Ø90 mm) were uniformly coated with 20 μL of the 24hFL, CFF of the 24hFL, or BS of the 24hFL. Subsequently, a plug (Ø7 mm) from the agar of each of the fungi, which were incubated for 7 days, was punched and separately placed at the center of a fresh PDA medium. Then, a Petri dish “sandwich” was made (Jiang et al., 2014) using the PDA medium inoculated with a pathogenic fungus on the bottom and a *B. subtilis* CF-3 liquid-coated LB agar medium on the top. The set of two plates was sealed with parafilm and incubated at 28°C. LB plates coated with distilled water (for fermentation liquids and CFF of the 24hFL) or TPS (for BS of the 24hFL) were used as negative controls. The mycelial diameter (mm) of the fungi was measured after 7 days. There were three replicates for each treatment, and the experiment was repeated three times.

The inhibition rate of mycelial growth *in vitro* (R) was calculated using the equation:

(1) $$\begin{array}{r} R\left(\%\right)=\frac{D_{1}-D_{2}}{D_{1}-D_{0}}\times 100 \end{array}$$

where *R* is the percent of inhibition of mycelial extension; *D*_1_ is the mycelial diameter (mm) of the negative control set; *D*_2_ is the mycelial diameter of the treated set, including the size of the fungal agar plug (mm); and *D*_0_ is the original mycelial diameter (Ø7 mm) of the punched fungal agar plug.

### HS-SPME-GC-MS analysis of the dynamic change of VOCs from *B. subtilis* CF-3 during fermentation and GC-MS conditions

We analyzed the VOCs in the fermentation liquids (from the 12hFL to the 96hFL) of *B. subtilis* CF-3. Peak area was used to evaluate the dynamic quantitative changes of VOCs during fermentation. The conical flasks loaded with the fermentation liquid were taken out one by one in 12-h intervals and prepared for the extraction process. The flasks were sealed with silicone septa and aluminum caps, placed in a temperature-controlled water bath for 30 min at 30°C to equilibrate, and then maintained in water baths at 39°C. To extract VOCs secreted by *B. subtilis* CF-3, three different kinds of extraction fibers were used: 85-μm polyacrylate (PA; Supelco, Bellefonte, PA, USA) fiber for extracting strongly polar compounds, 100-μm polydimethylsiloxane (PDMS; Supelco) fiber for extracting low-molecular weight VOCs, and 7-μm PDMS (Supelco) fiber for extracting high-molecular weight VOCs. Before analysis, the fibers were preconditioned as follows: the 85-μm PA fiber was heated at 280°C for 1 h, the 100-μm PDMS fiber was heated at 250°C for 30 min, and the 7-μm PDMS fiber was heated at 320°C for 1 h. The fibers were inserted into the conical flasks through the silicone pads, fixed at about 1 cm above the surface of the liquid, and exposed to headspace for 41 min at 39°C to extract the VOCs. One flask of fresh LB liquid medium was used as a control and processed under the same conditions to eliminate the substances volatilized from the liquid medium. Subsequently, the fibers were removed from the flasks and immediately inserted into the injection port of the GC-MS apparatus for analysis. The GC-MS conditions and procedures were selected as previously described (Gao H. Y. et al., 2017): when one compound was identified by multiple extraction fibers simultaneously, the conditions that yielded the highest probability were adopted. Each fermentation liquid was analyzed in triplicate.

### Dynamic change in rates of fungal pathogen inhibition by *B. subtilis* CF-3 VOCs during fermentation *in vitro*

Fermentation liquids of *B. subtilis* CF-3 (from the 12hFL to the 96hFL; 20 μL) were uniformly spread onto LB agar plates (Ø90 mm). Subsequently, a plug (Ø7 mm) from the agar of each of the fungi, which were incubated for 7 days, was punched and placed onto the center of a fresh PDA plate. A sandwich was made, with the PDA medium with the fungi on the bottom and the *B. subtilis* CF-3 liquid-coated LB agar on the top. The set of two plates was sealed with parafilm and incubated at 28°C. Plate sets of the fungal pathogen PDA medium on the bottom and the LB plate coated with distilled water on the top were used as negative controls and incubated under the same conditions. The mycelial diameter (mm) of fungal pathogens was measured after 7 days. The inhibition rate was calculated by Equation 1. There were three replicates for each treatment, and the experiment was repeated three times.

### Evaluation of inhibitory effects of single identified VOCs on fungal pathogens *in vitro*

Compounds were selected based on the correlation between the dynamic change of peak area in one compound during fermentation and the dynamic inhibition rate of the VOCs from *B. subtilis* CF-3 on fungal pathogens during fermentation through bivariate correlation analysis, and those that showed significant (*P* < 0.05) positive correlations were selected for the subsequent experiments. The selected compounds (technical grade) were purchased from Sinopharm Chemical Reagent Co., Ltd. (Shanghai, China). The compounds were serially diluted six times by 10-fold dilution in dimethyl sulfoxide. All purchased original reagents that were in solid state under normal conditions were dissolved in advance and diluted to 1 mol/L using dimethyl sulfoxide. The mycelial inhibition rates of fungal pathogens were evaluated as the inhibition activity. The reagent liquid (20 μL) was uniformly spread onto a water-agar plate (18 g agar diluted with distilled sterile water to 1 L). Subsequently, plugs (Ø7 mm) from the agars on which the fungi were incubated were punched and placed onto the center of fresh PDA media, and sandwiches of the two plates was sealed together with parafilm, as described previously. Water-agar plates not coated or coated with dimethyl sulfoxide were incubated and used as controls. The mycelial extension (mm) of the fungi was measured after 7 days. The inhibition rate of mycelial growth was calculated by Equation 1. In addition, the half maximal effective concentration (EC_50_) was calculated. There were three replicates for each treatment, and the experiment was repeated three times.

### Inhibitory effects of single identified VOCs and the 24hFL of *B. subtilis* CF-3 on fungal pathogens *in vivo*

Two *in vivo* experiments on peaches and litchi fruit were performed. For the peaches, a uniform wound (10 mm in diameter × 5 mm deep) was made at the equator of each peach using a sterilized puncher. Subsequently, a spore suspension of *M. fructicola* (20 μL) was applied to the wound sites at the same depth and air-dried. For litchi fruit, a spore suspension of *C. gloeosporioides* (20 μL) was directly injected into the flesh (2 mm deep) using a sterile syringe, and the fruit was air-dried. After inoculation, the fruits were placed on sterile plastic trays (4 × 3 wells, each 400 × 280 mm; each well for 1 peach or 2 litchi fruit), and the first and the fourth well in the middle row were left empty for applying VOCs. Afterwards, two filter papers (Ø90 mm) were placed onto the two empty wells, and 200 μL of the original reagent liquid of each single identified VOCs or of the 24hFL of *B. subtilis* CF-3 was dripped onto filter paper. All purchased original reagents that were in solid state under normal conditions were dissolved in advance and diluted to 1 mol/L using dimethyl sulfoxide. The trays were then put into a carton (450 × 350 × 100 mm). After being sealed, the carton was packed into polyethylene packaging bags and stored at room temperature (25°C). Cartons with filter papers that were not soaked (negative control for original reagent liquid) or soaked with distilled water (negative control for the 24hFL of *B. subtilis* CF-3) or dimethyl sulfoxide (negative control for dissolved solid reagents) were also stored under the same conditions. The mycelial diameter (mm) and healthy fruit rate (%) were evaluated every day at the same time up to the fifth day after inoculation. Considering the instability of VOCs, the fruits were discarded after daily evaluation and new cartons were opened the next day. There were three replicates of 10 peaches and 20 litchi fruit for each treatment, and the experiment was repeated three times.

The inhibition rate of mycelial growth *in vivo* (R_f_) was calculated using the equation:

(2) $$\begin{array}{r} R_{f}\left(\%\right)=\frac{D_{f1}-D_{f2}}{D_{f1}}\times 100 \end{array}$$

where *R*_*f*_ is the percent of inhibition of mycelial extension, *D*_*f*1_ is the mycelial diameter (mm) of the negative control set, and *D*_*f*2_ is the mycelial diameter (mm) of the treated set.

### Statistical analysis

As one of the objectives of this study was to assess the dynamic change in VOCs, the differences in the volatile profiles during fermentation were analyzed using analysis of variance (ANOVA), using the SPSS 19.0 software (SPSS Inc., Chicago, IL, USA). Duncan's *post-hoc* test was applied to compare the mean values of the VOCs. A significance level of *P* < 0.05 was used to determine the significant differences of the content of VOCs produced after different fermentation times. Mean values with standard deviations were reported.

Additionally, analysis of the correlations between the peak areas of identified VOCs and the inhibition rates was carried out by bivariate correlation analysis and calculated using the SPSS 19.0 software. The EC_50_ of single VOCs was calculated using GraphPad Prism 6.01 (GraphPad Software, Inc., La Jolla, CA, USA). All graphs were produced by Origin Pro 8.5 (OriginLab Corp., Northampton, MA, USA).

## Results

### Inhibitory effects of VOCs produced by *B. subtilis* CF-3 on fungal pathogens *in vitro*

**Figure 1:**
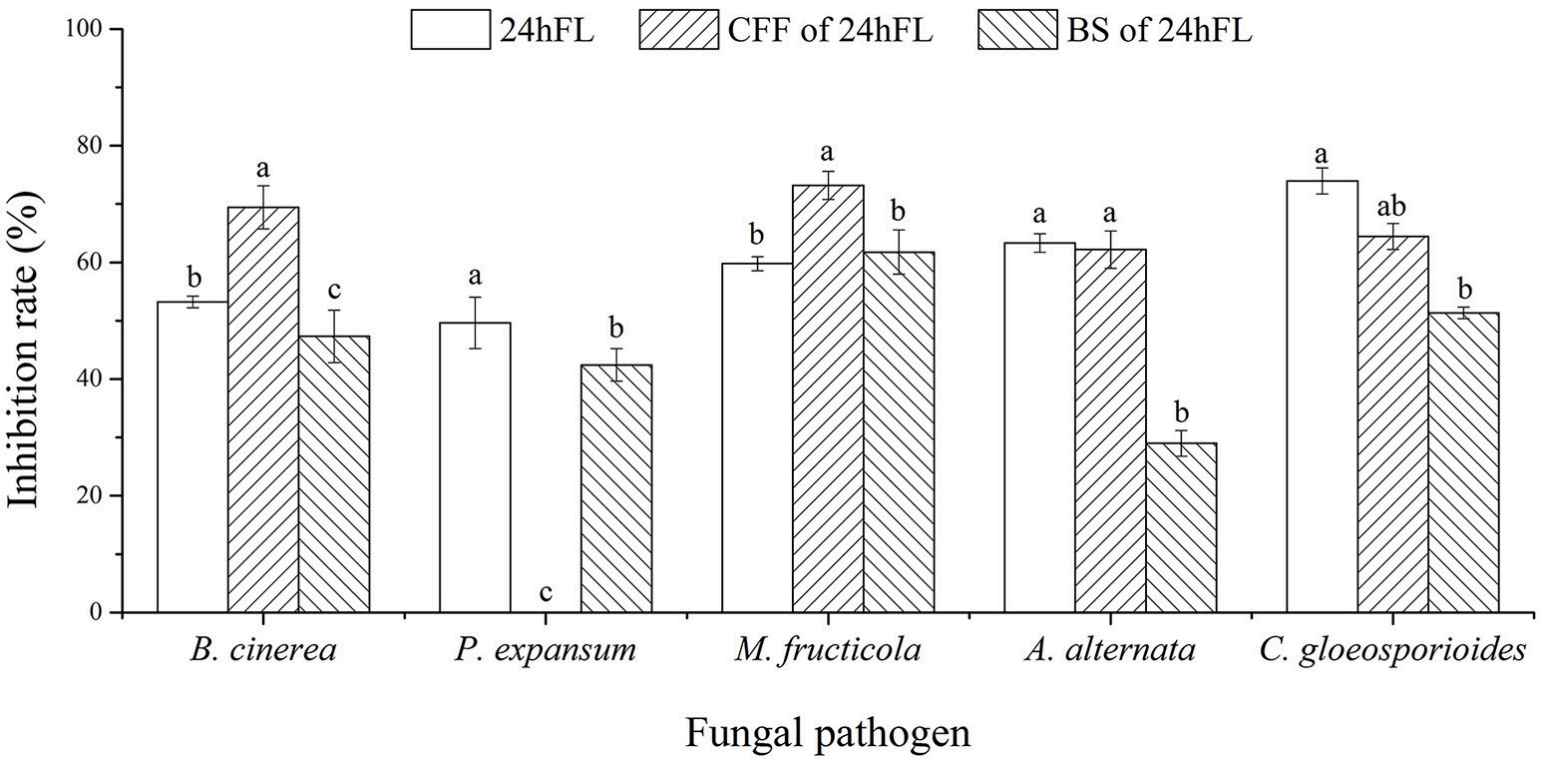
Inhibitory effects of VOCs from *B. subtilis* CF-3 fractions on fungal pathogens *in vitro*. “24hFL” indicates the 24-h fermentation liquid of *B. subtilis* CF-3, “CFF of 24hFL” indicates the cell-free filtrate of the 24-h fermentation liquid of *B. subtilis* CF-3, and “BS of 24hFL” indicates the bacterial suspension of the 24-h fermentation liquid of *B. subtilis* CF-3. Each column represents the mean value from three independent experiments, and vertical bars represent the standard errors of the means for each treatment. Different letters represent significant differences (*P* < 0.05) in the inhibition rates between different treatments on the same fungi.

**Figure 2:**
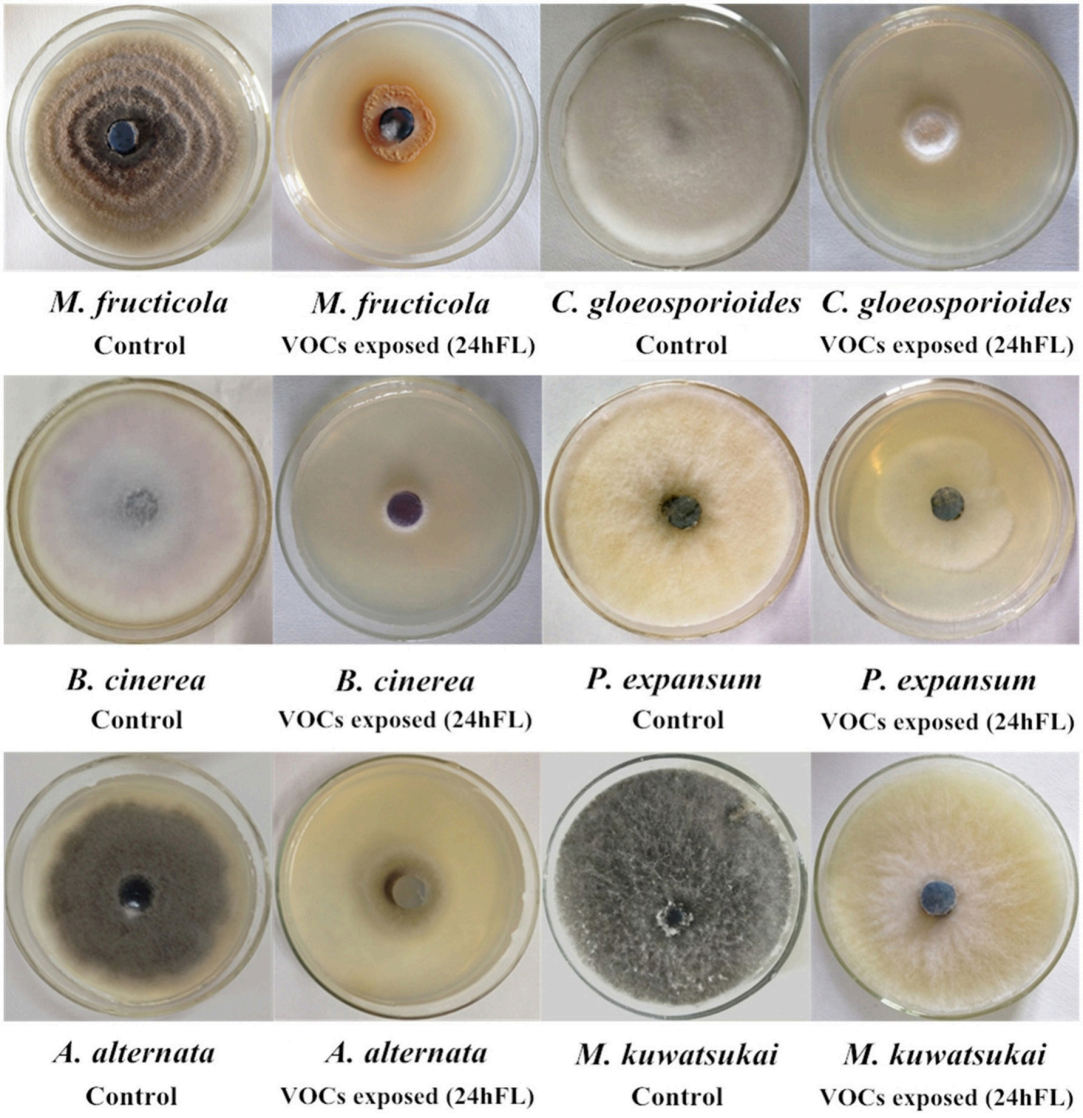
Mycelial growth of common fruit fungal pathogens exposed to VOCs from 24-h fermentation liquid (24hFL) of *B. subtilis* CF-3 and control *in vitro*.

To explore the biocontrol effects of VOCs produced by *B. subtilis* CF-3 on different common fungal pathogens, the inhibition rate of VOCs was evaluated in three different fractions: the 24hFL, CFF of the 24hFL, and BS of the 24hFL. The effects of VOCs from the 24hFL on *M. fructicola* and *C. gloeosporioides* had already been estimated in a previous study (Gao H. Y. et al., 2017). The results demonstrated that most of the treatments had strong antifungal abilities against most selected fungal pathogens except on *M. kuwatsukai*, which was not inhibited by any treatment. Additionally, *P. expansum* was not inhibited by the VOCs of the CFF of the 24hFL. As shown in Figures 1, 2 the antifungal effects depended on the pathogens and treatment. The VOCs of the 24hFL exhibited the highest mean inhibition rate against all tested fungal pathogens in all treatments (59.97%), and the mean rates for the CFF and BS of the 24hFL were 53.84 and 46.36%, respectively. In the present study, almost all collected fungal pathogens were inhibited, indicating that *B. subtilis* CF-3 has a relatively broad antifungal spectrum; thus, it is necessary to identify the key inhibitory VOCs produced by *B. subtilis* CF-3. Among the tested pathogens, *M. fructicola* was the most susceptible: the mean inhibition rate of the three treatments was 64.91%. Furthermore, *C. gloeosporioides* was strongly inhibited, with a mean rate of 63.22%. These *two* fungi are common fruit fungal pathogens (Munir et al., 2016; Fischer et al., 2017), which is why they have been chosen as targets in the subsequent experiments.

### Changes in VOC production at different fermentation durations, their inhibitory effects on *M. fructicola* and *C. gloeosporioides*, and the correlation between them

In this study, 74 different compounds volatilized in the liquids of *B. subtilis* CF-3 at different fermentation times were identified using HS-SPME-GC-MS (Table 1, Table S1): 15 alcohols, 18 ketones, 4 pyrazines, 4 esters, 10 acids, 5 phenols, 3 hydrocarbons, 3 amines, 2 aldehydes, 5 ethers, and 5 other components. We used three different types of extraction fibers to extract the VOCs because of their different molecular polarity. Among the fibers, the 85-μm PA fiber extracted most kinds of VOCs (38 different compounds) and with higher probability. The largest number of possible VOCs (62 different compounds) was obtained from the 48hFL. As shown in Table 1, the content and number of each class did not show correlation with fermentation duration. The trend in the dynamic total peak area was as follows: during the initial stage, the total peak area of all possible VOCs rose to 5004596 in the 12hFL and decreased to 4620461 in the 24hFL; subsequently, the peak area sharply increased to 11527435 in the 36hFL and reached its peak, 19971460, in the 48hFL, after which it gradually declined in the second half of the fermentation period (48hFL−96hFL). Moreover, there was no significant difference (*P* > 0.05) in the amount of total possible VOCs between the 12hFL and 24hFL, and between the 72hFL and 84hFL.

**Table 1:** The main classes of VOCs produced by *B. subtilis* CF-3 after different fermentation durations.

**Class**	**Relative content (%)**							
	**12hFL**	**24hFL**	**36hFL**	**48hFL**	**60hFL**	**72hFL**	**84hFL**	**96hFL**
Alcohols	15.03 ± 0.38 d	11.60 ± 0.34 e	12.64 ± 0.28 c	9.63 ± 0.12 a	24.41 ± 0.48 b	5.95 ± 0.15 g	8.24 ± 0.29 f	5.26 ± 0.23 h
Number of alcohols	13	12	13	14	14	14	14	13
Ketones	22.54 ± 1.28 c	18.03 ± 0.92 d	15.62 ± 1.09 b	10.56 ± 0.35 a	11.15 ± 0.68 d	15.04 ± 0.82 e	16.55 ± 1.25 e	7.40 ± 0.52 f
Number of ketones	14	13	9	15	14	12	13	9
Pyrazines	0.55 ± 0.05 b	0.05 ± 0.02 b	0.35 ± 0.12 b	3.50 ± 0.73 a	0.07 ± 0.02 b	0.09 ± 0.03 b	0 b	0 b
Number of pyrazines	3	1	2	3	1	1	0	0
Esters	2.68 ± 0.52 cd	3.68 ± 0.86 c	8.99 ± 1.92 a	3.00 ± 0.49 b	1.40 ± 0.28 cd	0.33 ± 0.08 d	0.81 ± 0.29 d	0.30 ± 0.11 d
Number of esters	3	2	3	3	1	1	2	1
Acids	17.06 ± 2.93 b	13.53 ± 2.01 bc	4.30 ± 0.98 cd	10.66 ± 1.87 a	4.03 ± 0.51 de	6.22 ± 0.69 de	7.37 ± 1.28 de	7.83 ± 0.77 e
Number of acids	5	4	5	9	6	3	5	3
Phenols	8.49 ± 2.35 ab	13.71 ± 3.89 a	4.39 ± 1.52 ab	2.96 ± 0.68 a	4.51 ± 1.25 b	2.87 ± 0.48 c	2.72 ± 0.92 c	2.91 ± 1.04 c
Number of phenols	4	3	4	5	3	3	3	3
Hydrocarbons	0 c	0 c	3.81 ± 0.95 b	3.42 ± 0.52 a	1.01 ± 0.18 c	0 c	1.50 ± 0.32 c	0 c
Number of hydrocarbons	0	0	1	3	2	0	1	0
Amines	15.84 ± 2.09 b	0.14 ± 0.03 c	0.49 ± 0.15 c	12.86 ± 2.94 a	1.69 ± 0.39 c	0.60 ± 0.16 c	0.22 ± 0.09 c	0.39 ± 0.04 c
Number of amines	2	1	1	2	2	1	1	1
Aldehydes	2.17 ± 0.68 c	7.73 ± 1.29 a	1.67 ± 0.38 b	0.61 ± 0.13 c	1.60 ± 0.27 c	2.11 ± 0.54 cd	1.44 ± 0.20 c	1.28 ± 0.44 c
Number of aldehydes	2	2	2	2	2	2	2	2
Ethers	12.98 ± 2.23 c	17.71 ± 2.96 bc	6.63 ± 0.93 bc	9.57 ± 1.38 a	13.70 ± 2.40 b	27.05 ± 3.95 b	18.99 ± 3.29 c	15.72 ± 2.37 d
Number of ethers	3	4	3	3	4	4	3	2
Others	2.66 ± 0.39 e	13.77 ± 2.67 de	41.11 ± 7.93 b	33.23 ± 5.23 a	36.44 ± 8.30 c	39.74 ± 6.09 d	42.17 ± 4.94 d	58.89 ± 6.82 d
Number of others	2	2	4	3	2	2	4	2
Total peak area of all possible VOCs	5004596 ± 425236 d	4620461 ± 521412 d	11527435 ± 796523 b	19971460 ± 841253 a	7622145 ± 532115 c	3597881 ± 501027 e	3179336 ± 489365 e	1961563 ± 141255 f
Number of all possible VOCs	51	44	47	62	51	43	48	36

*The compounds possibly generated by CF-3 were identified by comparing their mass spectra to those in the NIST Mass Spectral Library (probability-based match >85%). Values are expressed as means of the relative content of each class (n = 3). To express the variation of the VOCs more accurately, peak area (relative content (%) × total peak area of all possible VOCs) was used in ANOVA. The different letters represented significant differences (P < 0.05) between the B. subtilis CF-3 fermentation liquids for different fermentation durations*.

Alcohols and ketones were the two dominant classes of VOCs produced by *B. subtilis* CF-3, amounting to 33 different compounds. In total, 15 kinds of alcohols were identified in this study. The number of volatilized alcohols was constant at approximately 13 kinds in all fermentation liquids, regardless of fermentation duration. The relative content of alcohols was 15.03% in the 12hFL, and it gradually increased to the 60hFL, when it peaked at 24.41%; afterwards, it gradually decreased. During the whole fermentation period, 18 ketones were identified. Fourteen were identified in the 12hFL, after which the number declined and reached the minimum (9 different ketones) in the 36hFL. However, the number of ketones rapidly rose after the 36hFL and peaked at 15 in the 48hFL, after which it gradually declined. At first, the relative content of ketones was 22.54% in the 12hFL and gradually decreased, reaching 10.56% in the 48hFL. Afterwards, the content gradually increased until it reached 16.55% in the 84hFL; in the 96hFL, it dropped to the minimum of 7.40%. Furthermore, 10 acids were identified in this study. In the early stage, the number of identified acids was about 5, after which the highest number of different volatilized acids (9 kinds) was identified in the 48hFL. In the mid and late fermentation periods, the number of different acids decreased. In the 12hFL and 48hFL, but not the 36hFL, the relative content of acids was relatively high. Subsequently, the relative content decreased. Among the VOCs, 5 identified compounds were phenols. The number of volatilized phenols was constant at about 4 kinds during the entire fermentation. The highest relative content of phenols (13.71%) was identified in the 24hFL. Furthermore, all fermentation liquids, regardless of fermentation duration, contained only two volatilized aldehydes, benzaldehyde and 3-methylbutanal. The relative content of aldehydes increased at first and peaked in the 24hFL at 7.73%, after which it gradually decreased. Of these two aldehydes, benzaldehyde occupied the larger portion. In addition, 2 unsorted compounds were identified in this study: methoxy-phenyl-oxime and benzothiazole. As the fermentation progressed, the relative content of methoxy-phenyl-oxime increased, peaking at 58.68% in the 96hFL, wherein it occupied the largest portion of unsorted compounds. Anisole, as the ether most commonly identified in this study, occupied a large proportion among the VOCs and was mostly produced in the 60hFL. Finally, the major VOCs produced by CF-3 that were identified at different fermentation times are presented in Table 2: most of the major VOCs, i.e., (*S*)-1-octanol, benzoic acid, 2,4-di-*tert*-butylthiophenol, benzaldehyde, and benzothiazole, were already produced by the 24hFL. As the fermentation progressed, the quantity of the major VOCs gradually decreased.

**Table 2:** The components of main VOCs produced by *B. subtilis* CF-3 after different fermentation durations.

**Possible VOC**	**Relative content (%)**							
	**12hFL**	**24hFL**	**36hFL**	**48hFL**	**60hFL**	**72hFL**	**84hFL**	**96hFL**
(*S*)-1-octanol	0.91 ± 0.28 b	3.27 ± 0.97a	0.47 ± 0.13 b	0.24 ± 0.09*b*	0.74 ± 0.21 b	0.33 ± 0.08 c	0.09 ± 0.03 c	0.09 ± 0.05 c
Benzoic acid	2.53 ± 0.69 c	9.16 ± 1.30 a	1.77 ± 0.12 b	0.39 ± 0.05 cd	1.24 ± 0.34 c	1.12 ± 0.16 de	0.69 ± 0.17 e	1.28 ± 0.27 e
2,4-di-*ter*t-butylthiophenol	3.25 ± 0.47 b	12.32 ± 2.36 a	2.16 ± 0.59 b	1.17 ± 0.27 b	2.62 ± 0.36 b	0.60 ± 0.14 c	0.47 ± 0.11 c	0 c
Benzaldehyde	1.85 ± 0.68 c	7.24 ± 1.22 a	1.40 ± 0.21 b	0.44 ± 0.08 cd	1.15 ± 0.32 cd	1.75 ± 0.17 cde	1.22 ± 0.27 de	1.16 ± 0.36 e
3-methylbutanal	0.32 ± 0.07 bc	0.49 ± 0.11 ab	0.26 ± 0.08 a	0.17 ± 0.06 a	0.45 ± 0.13 a	0.36 ± 0.10 bcd	0.22 ± 0.04 cd	0.13 ± 0.03 d
Anisole	12.54 ± 2.85 ab	11.59 ± 2.38 bc	5.88 ± 1.07 ab	4.02 ± 0.95 ab	11.27 ± 3.14 a	18.35 ± 3.36 ab	18.05 ± 2.87 b	15.34 ± 1.63 c
Methoxy-phenyl-oxime	1.41 ± 0.28 d	9.23 ± 3.36 cd	39.04 ± 7.35 a	13.03 ± 4.82 b	34.95 ± 7.36 b	37.56 ± 10.28 c	37.77 ± 8.36 c	58.68 ± 6.21 c
Benzothiazole	1.25 ± 0.36 cd	4.54 ± 1.82 a	1.11 ± 0.17 b	0.55 ± 0.08 bc	1.49 ± 0.36 bc	2.17 ± 0.38 bc	0.31 ± 0.08 d	0.21 ± 0.09 d
Total peak area of all possible VOCs	5004596 ± 425236 d	4620461 ± 521412 d	11527435 ± 796523 b	19971460 ± 841253 a	7622145 ± 532115 c	3597881 ± 501027 e	3179336 ± 489365 e	1961563 ± 141255 f

*The compounds possibly generated by CF-3 were identified by comparing their mass spectra to those in the NIST Mass Spectral Library (probability-based match >85%). Values are expressed as means of the relative content of each compound (n = 3). To express the variation of the VOCs more accurately, peak area (relative content (%) × total peak area of all possible VOCs) was used in ANOVA. The different letters represented significant differences (P < 0.05) between the B. subtilis CF-3 fermentation liquids for different fermentation durations*.

**Figure 3:**
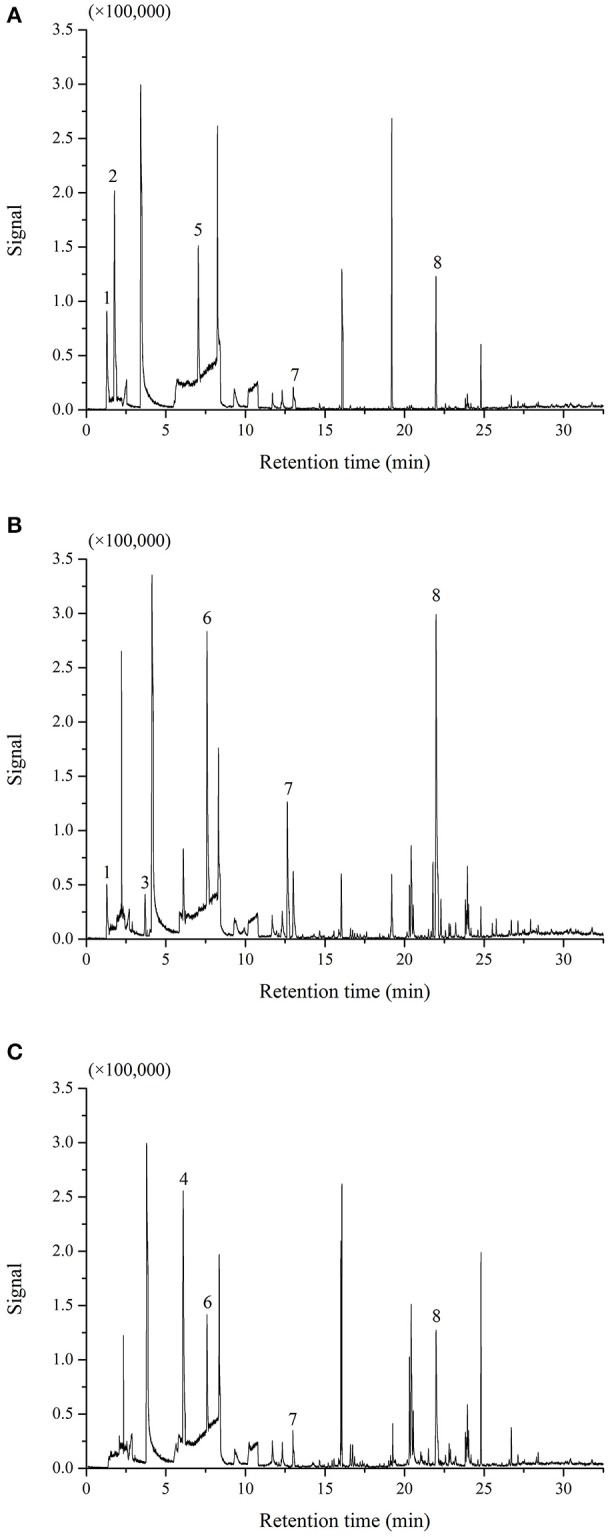
Chromatograms of VOCs produced by the 24-h fermentation liquid (24hFL) extracted by 7-μm PDMS **(A)**, 85-μm PA **(B)**, and 100-μm PDMS **(C)**. The main VOCs were noted as (1) (*S*)-1-octanol, (2) benzaldehyde, (3) 3-methylbutanal, (4) benzoic acid, (5) methoxy-phenyl-oxime, (6) anisole, (7) benzothiazole, and (8) 2,4-di-*ter*t-butylthiophenol. The remaining peaks represent other minor VOCs or the fibers and atmosphere.

The rates of *M. fructicola* and *C. gloeosporioides* inhibition by the VOCs produced by *B. subtilis* CF-3 were measured at different fermentation times and listed in Table 3. The inhibition rate of *M. fructicola* reached its peak of 73.46 ± 3.88% by 24hFL, and there was a subsequent overall declining trend in the inhibition that included one rebound by 96hFL. The inhibition rate of *C. gloeosporioides* first reached its peak of 63.63 ± 4.69% by 24hFL, then decreased gradually except aside from a rebound by 60hFL. Although the most VOCs were identified in 48hFL, the VOCs from 24hFL showed the strongest inhibitory effects among all the liquids on both fungal pathogens. The GC-MS chromatograms of VOCs produced by 24hFL are shown in Figure 3.

**Table 3:** Rates of *M. fructicola* and *C. gloeosporioides* inhibition by VOCs produced by *B. subtilis* CF-3 at different fermentation times.

**Fungal pathogen**	**Inhibition rate (%) on fungal pathogens**							
	**12hFL**	**24hFL**	**36hFL**	**48hFL**	**60hFL**	**72hFL**	**84hFL**	**96hFL**
*M. fructicola*	69.78 ± 3.17ab	73.46 ± 3.88a	64.65 ± 4.13bc	59.94 ± 5.54c	51.50 ± 3.52d	46.85 ± 4.42d	33.43 ± 5.05e	38.48 ± 4.68e
*C. gloeosporioides*	59.11 ± 5.39a	63.63 ± 4.69a	57.30 ± 4.10a	56.64 ± 3.81a	59.88 ± 3.98a	43.11 ± 2.33b	42.50 ± 5.11b	22.27 ± 4.08c

*The different letters represent significant differences (P < 0.05) in inhibition rates between the different B. subtilis CF-3 fermentation liquids on the same fungal pathogen*.

Moreover, correlations between the peak area of VOCs produced by *B. subtilis* CF-3 and the inhibition rates on *M. fructicola* and *C. gloeosporioides* were analyzed by bivariate correlation analysis. The key compounds that had significant (*P* < 0.05) positive correlation with the inhibition rate are listed in Table 4. We observed significant (*P* < 0.05) positive correlations between the amounts of produced 2,4-di-*tert*-butylphenol, 1-octanol, and benzothiazole and the inhibition rates against both *M. fructicola* and *C. gloeosporioides*. Additionally, benzoic acid and benzaldehyde were significantly (*P* < 0.05) positively correlated with the inhibition rate against *M. fructicola*, and anisole and 3-methylbutanal were significantly (*P* < 0.05) positively correlated with the inhibition rate against *C. gloeosporioides*. Those VOCs that exhibited significant (*P* < 0.05) positive correlations with inhibition rates were chosen as target VOCs in the following experiment.

**Table 4:** The Pearson correlation coefficients of single compounds whose levels had significant positive correlations (*P* < 0.05) with the rates of *M. fructicola* and *C. gloeosporioides* inhibition.

**Volatile compound**	**With dynamic rate of** ***M. fructicola*** **inhibition**		**With dynamic rate of** ***C. gloeosporioides*** **inhibition**	
	**Pearson correlation coefficient**	***P*****-value**	**Pearson correlation coefficient**	***P*****-value**
2,4-di-*tert*-butylphenol	0.817	0.013	0.748	0.033
1-octanol	0.799	0.017	0.730	0.040
Benzothiazole	0.793	0.019	0.786	0.021
Benzoic acid	0.782	0.022	-	ns
Benzaldehyde	0.759	0.029	-	ns
Anisole	-	ns	0.712	0.048
3-methylbutanal	-	ns	0.795	0.018

*“-” means this compound showed no significant correlation with the dynamic inhibition rate, and “ns” means not significant (P > 0.05)*.

### Inhibitory effects of the identified VOCs on fungal pathogens

#### Inhibitory effects of single identified VOCs on fungal pathogens *in vitro*

**Figure 4:**
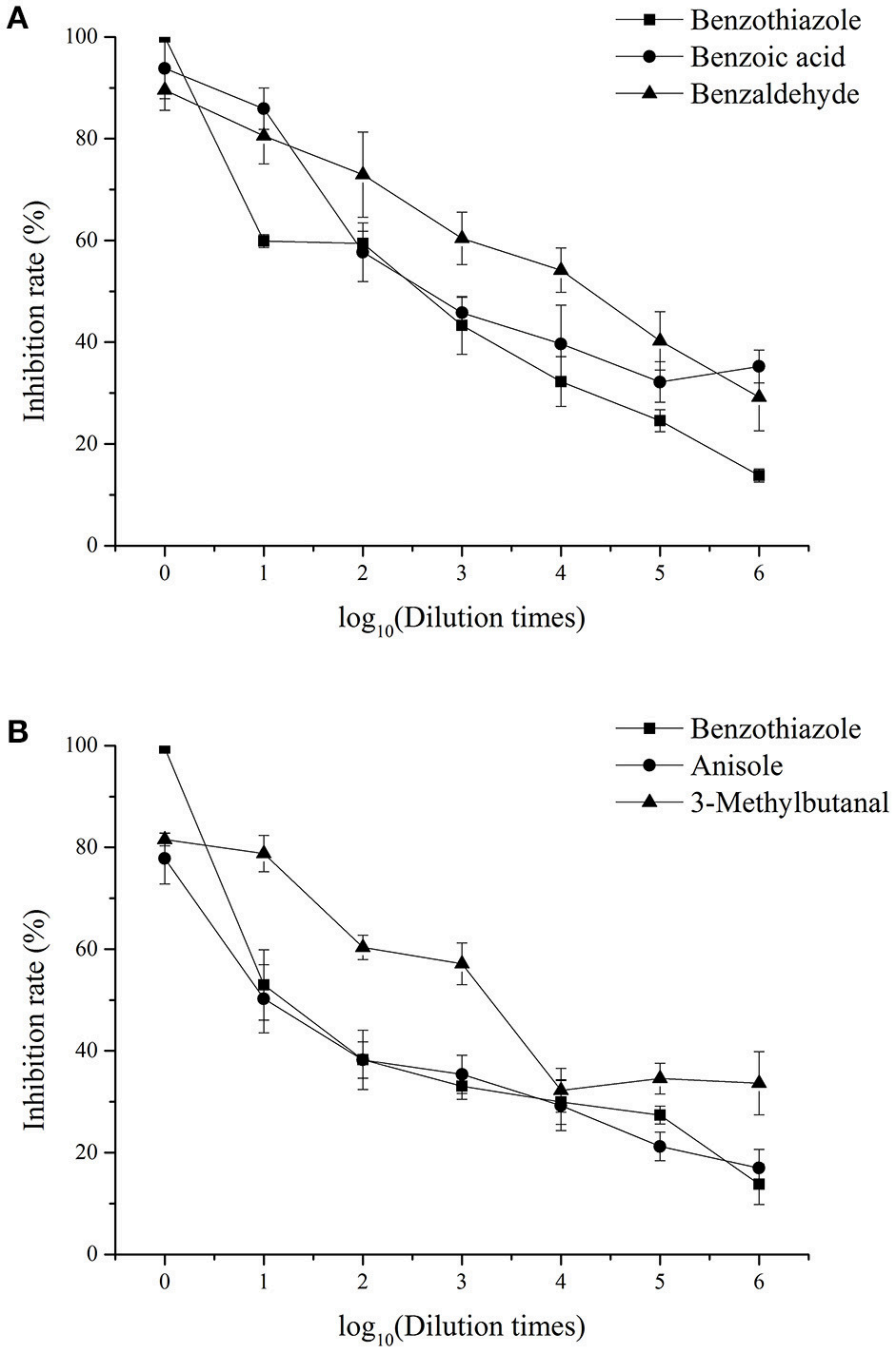
Inhibitory effects of single identified VOCs on *M. fructicola* **(A)** and *C. gloeosporioides* **(B)** after different dilution times *in vitro*. Each spot represents the mean value from three independent experiments, and the vertical bars represent the standard errors of the means for each treatment.

The inhibition rate of single identified VOCs on *M. fructicola* and *C. gloeosporioides* after different dilution times is shown in Figure 4, while the EC_50_ of the VOCs are shown in Table 5. The EC_50_ values of 2,4-di-*tert*-butylphenol and 1-octanol on the two selected fungal pathogens were reported previously (Gao H. Y. et al., 2017). Among the selected compounds, 1-octanol showed the strongest inhibitory effect against *M. fructicola*, and its EC_50_ was 4.77 × 10^−5^ mol/L. Furthermore, 2,4-di-*tert*-butylthiophenol (EC_50_ = 9.90 × 10^−4^ mol/L) and benzaldehyde (EC_50_ = 6.70 × 10^−4^ mol/L) exhibited strong inhibitory effects as well. Additionally, 3-methylbutanal showed the strongest inhibitory effect on *C. gloeosporioides*, with an EC_50_ of 7.67 × 10^−3^ mol/L, and 2,4-di-*tert*-butylthiophenol showed a strong inhibitory effect (EC_50_ = 1.26 × 10^−2^ mol/L) as well; the remaining compounds exhibited relatively weak inhibitory effects.

**Table 5:** EC_50_ of different VOCs for inhibiting the mycelial growth of *M. fructicola* and *C. gloeosporioides*.

**Volatile compound**	***M. fructicola***	***C. gloeosporioides***
	**EC_50_ (mol/L)**	**EC_50_ (mol/L)**
Benzothiazole	2.29 × 10^−2^	0.36
Benzoic acid	1.46 × 10^−3^	nt
Benzaldehyde	6.70 × 10^−4^	nt
Anisole	nt	0.59
3-methylbutanal	nt	7.67 × 10^−3^

*“nt” means not tested in this study*.

#### Inhibitory effects of single identified VOCs and the 24hFL from *B. subtilis* CF-3 on fungal pathogens *in vivo*

**Figure 5:**
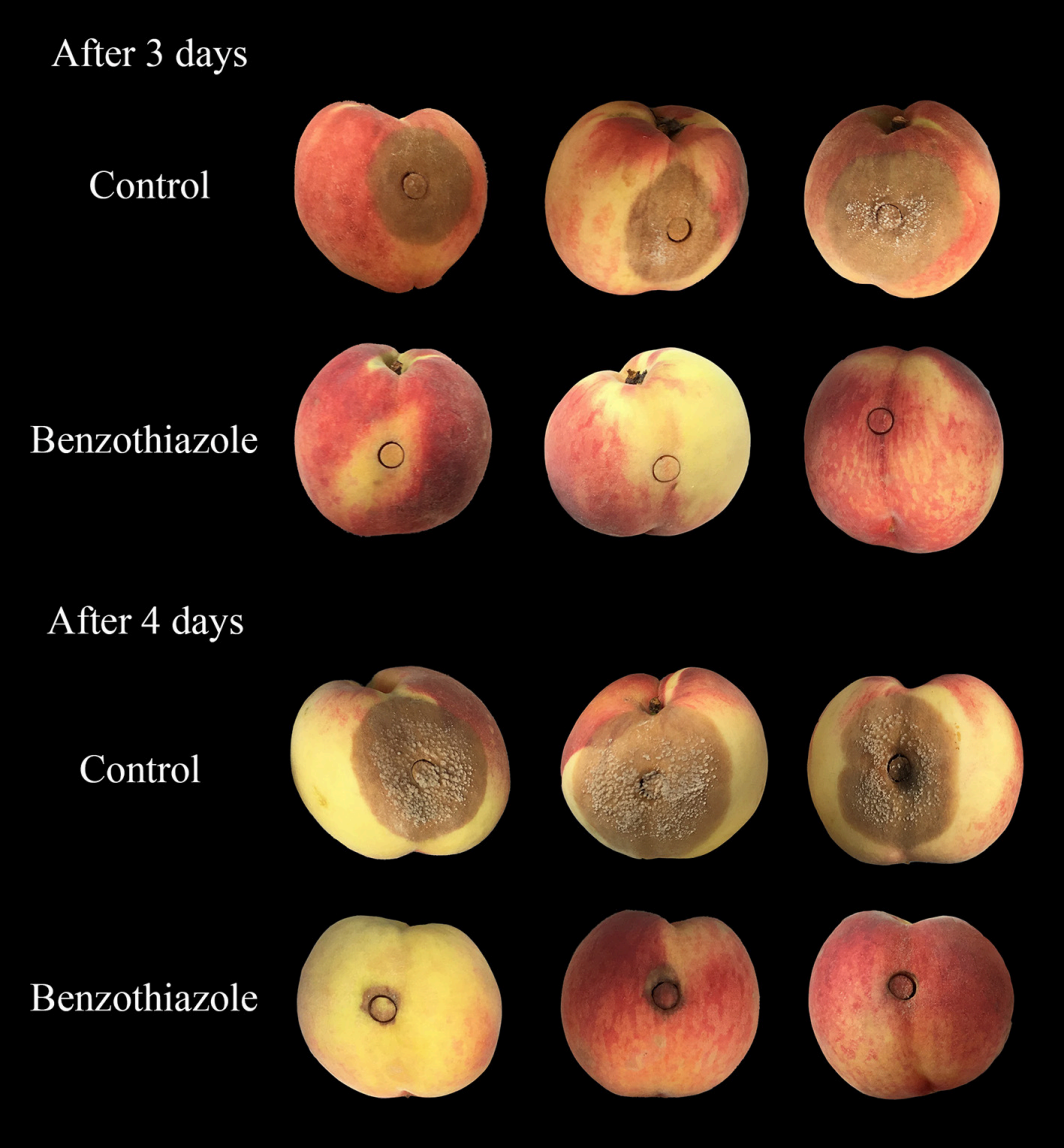
Effects of benzothiazole on *M. fructicola in vivo* after 3 and 4 days.

**Figure 6:**
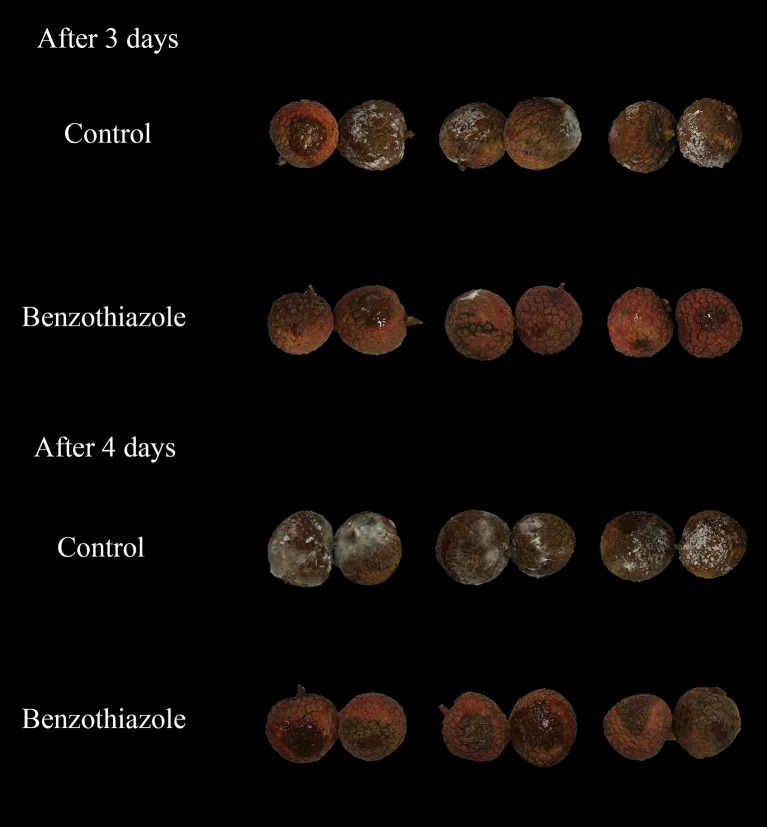
Effects of benzothiazole on *C. gloeosporioides in vivo* after 3 and 4 days.

**Figure 7:**
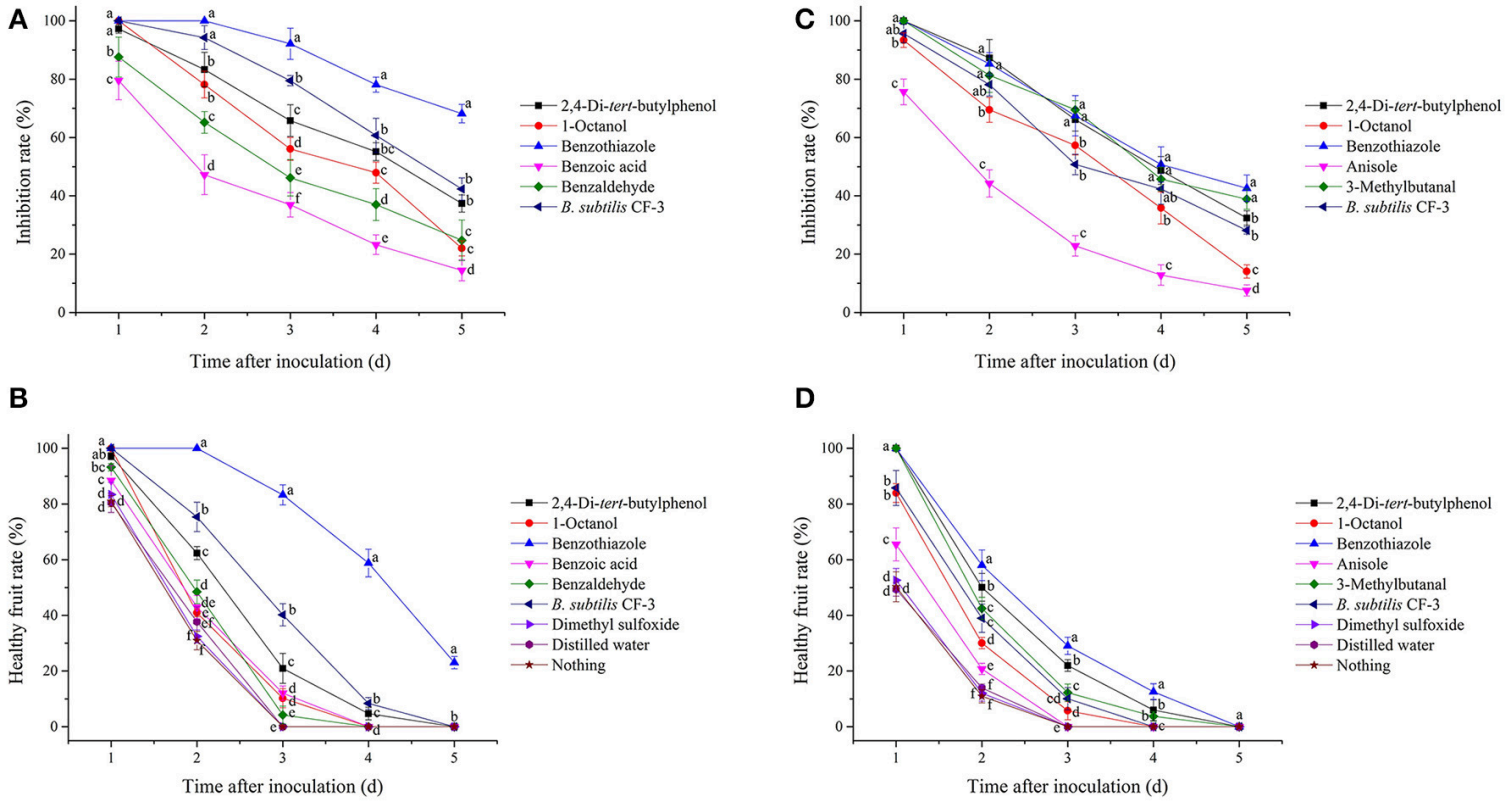
Inhibitory effects of single identified VOCs and the 24-h fermentation liquid (24hFL) of *B. subtilis* CF-3 on peaches **(A,B)** and litchi fruit **(C,D)** *in vivo*. Two indexes were evaluated: the mycelial inhibition rate **(A,C)** and the healthy fruit rate **(B,D)**. Each dot represents the mean of replicates from three independent experiments, and the vertical bars represent the standard errors of the means for each treatment. Different letters represent significant differences (*P* < 0.05) between different treatments on the same day.

In the present study, two indexes, the mycelial inhibition rate and the healthy fruit rate, were evaluated on both peaches and litchi fruit (Figures 5, 6). In peaches, benzothiazole treatment showed the strongest inhibitory effect on the mycelial growth among all treatments (Figure 7A). Moreover, benzothiazole treatment significantly increased the healthy fruit rate (Figure 7B), which was still at approximately 20% after 5-day storage at 25°C, while no fruit remained healthy in the other treatment groups after 5 days. The 24hFL from *B. subtilis* CF-3 showed relatively strong inhibitory effects *in vivo*, which were inferior only to those of benzothiazole. In litchi fruit, all compounds showed inhibitory effects on pathogen mycelial growth, and, among the compounds, anisole showed the weakest inhibitory effect (Figure 7C). Although there was no significant difference between the other treatments, benzothiazole treatment showed the strongest relative inhibitory effect. After storage at 25°C for 5 days, no fruit remained healthy in any of the treatment groups (Figure 7D).

## Discussion

Considering the risks the drug resistance of fungal pathogens and environmental pollution caused by the abuse of chemical fungicides present to human health, the need for both environmentally friendly and more effective methods for controlling fungal diseases in plants is increasing rapidly. Biological control has been widely regarded as a potential substitute for chemical control because of its high efficiency and environmental safety, and *B. subtilis* has been widely studied in this context. The main antifungal substances secreted by *B. subtilis* are enzymes and antibiotic lipopeptides, including surfactin, iturin, and fengycin (Ongena et al., 2007; Wang and Yeh, 2008; Liu et al., 2011; Torres et al., 2016). However, recent research has indicated that VOCs produced by *B. subtilis*, its primary and secondary metabolites, also have strong antifungal effects and application potential (Leelasuphakul et al., 2008; Arrebola et al., 2010; Zheng et al., 2013).

In a previous study, the VOCs produced by the *B. subtilis* CF-3 strain were isolated and identified from fermented bean curd (Gao et al., 2016). The results of the present study indicate that the VOCs produced by *B. subtilis* CF-3 have inhibitory effects on several common fungal pathogens *in vitro*. To clarify whether VOCs from the CFF or BS also have inhibitory effects on the tested fungal pathogens, the inhibition rates of the CFF and BS were also evaluated. The results demonstrated that the inhibitory effects of VOCs from the studied liquids differed depending on the given pathogen. This indicates that optimal treatments for different fungal pathogen infections will be different. Moreover, although the CFF and BS were separated from the 24hFL, the sum of the inhibition rates of these two component liquids did not equal the inhibition rate of the 24hFL, indicating that some interactions between the VOCs and the CF-3 strain that enhance the inhibition of fungal pathogens may exist. Although the 24hFL did not contain the majority of the VOCs, the VOCs from the 24hFL showed the strongest inhibitory effects among all fermentation liquids on both *M. fructicola* and *C. gloeosporioides*. The 24hFL contained more key inhibitory VOCs like (*S*)-1-octanol, benzoic acid, 2,4-di-*tert*-butylthiophenol, benzaldehyde, and benzothiazole (Table 2), and it showed a significant positive correlation with the inhibition rates (Table 4) compared to the other fermentation liquids.

Previous studies have classified the common VOCs secreted by microorganisms into alcohols, aldehydes, hydrocarbons, esters, ketones, organic acids, and other compounds (Wang et al., 2016). In the present study, three different extraction fibers were used, and the 85-μm PA fibers were shown to be optimal for extraction, since they extracted the most VOCs (38 different compounds) with a higher probability, in conformance with our previous study (Gao H. Y. et al., 2017). Based on the bivariate correlation analysis, the quantities of three VOCs, 2,4-di-*tert*-butylphenol, 1-octanol, and benzothiazole, showed a significant (*P* < 0.05) correlation with the rates of *M. fructicola* and *C. gloeosporioides* inhibition. Previous studies have reported that 2,4-di-*tert*-butylphenol has antifungal abilities against several fungi such as *Aspergillus niger* and *Penicillium chrysogenum* (Varsha et al., 2015; Belghit et al., 2016). Additionally, 2,4-di-*tert*-butylphenol, as a VOC produced by *Pseudomonas monteilii* PsF84, has been shown to inhibit the spore germination and hyphal growth of *Fusarium oxysporum* (Dharni et al., 2014). Our HS-SPME-GC-MS results showed relatively high 2,4-di-*tert*-butylphenol content among the VOCs, its relative content in the 24hFL being 12.32%. The calculation of EC_50_ also demonstrated that 2,4-di-*tert*-butylphenol had relatively strong inhibitory activity against the two selected fungal pathogens: 9.90 × 10^−4^ mol/L for *M. fructicola* and 1.26 × 10^−2^ mol/L for *C. gloeosporioides* (Gao H. Y. et al., 2017). These high values might explain why the 24hFL from *B. subtilis* CF-3 showed the strongest inhibitory effect despite not containing the majority of the VOCs. Moreover, in a previous study, 2,4-di-*tert*-butylphenol also showed a relatively strong inhibitory effect on mycelial growth of *M. fructicola* in an *in vivo* experiment, in which its inhibition rate remained at about 40% after 5-day incubation (Gao H. Y. et al., 2017). These results demonstrated that 2,4-di-*tert*-butylphenol, which was present at high levels in the fermentation liquids and showed strong inhibitory effects, is a key antifungal VOC produced by *B. subtilis* CF-3.

In addition to 2,4-di-*tert*-butylphenol, 1-octanol and benzothiazole also played important roles in fungal inhibition. 1-Octanol has been demonstrated to completely inhibit spore germination of *Penicillium camemberti* at low concentrations (Gillot et al., 2016). Benzothiazole and its derivatives are regarded as some of the most useful heterocyclic compounds and are widely used in medicinal chemistry for their effects (Keri et al., 2015). Benzothiazole showed the strongest inhibitory effect on both fungal pathogens and increased the healthy fruit rate in both peaches and litchi fruit in the *in vivo* experiment. VOCs released by *Bacillus velezensis* ZSY-1, among which benzothiazole had a much larger relative peak area than the other compounds, were shown to exhibit significant antifungal activity against *M. fructicola*, and benzothiazole has been demonstrated to be a promising bioagent for controlling fungal diseases such as gray mold and early blight on tomato fruit (Gao Z. F. et al., 2017). Additionally, benzothiazole, which was also found in the VOCs produced by fungi of the *Xylariaceae* family in olive trees, has been shown to play a significant role in reducing the mycelial expansion of *Colletotrichum acutatum* in olives (Landum et al., 2016). In the present study, benzothiazole did not show the strongest inhibitory effect on mycelial growth of fungal pathogens *in vitro*. However, it showed the strongest inhibitory effect *in vivo*. The results indicate that benzothiazole, which displayed prominent inhibitory effects *in vivo*, especially on *M. fructicola*, is a key antifungal VOC produced by *B. subtilis* CF-3. Besides benzothiazole, other VOCs showed different inhibitory effects in *in vivo* and *in vitro* experiments. This difference may not only be due to the direct inhibition of the growth of fungal mycelia, but also due to some VOCs stimulating the fruit to activate enzymes that improve the defense against fungal infections. 3-methylbutanal showed strong inhibitory effects on *C. gloeosporioides* in both the *in vivo* and *in vitro* experiments. In addition, it reportedly had an inhibitory effect on some fungal pathogens in other studies. Isovaleraldehyde (3-methylbutanal) was identified in the essential oil obtained from *Senecio graveolens* (*Compositae*), which showed inhibitory effects on the growth of *Candida albicans* (Perez et al., 1999).

The results of the present study indicate that 2,4-di-*ter*t-butylthiophenol and benzothiazole are two key inhibitory VOCs produced by *B. subtilis* CF-3. However, the mechanisms by which these two compounds inhibit the growth of *M. fructicola* and *C. gloeosporioides in vivo* and *in vitro* are still unclear and ought to be clarified in future studies.

## Conclusion

The VOCs produced by *B. subtilis* CF-3 showed antifungal activity against several common fruit fungal pathogens. *M. fructicola* and *C. gloeosporioides* were the two most inhibited fungi. Using HS-SPME-GC-MS, 74 probable VOCs were identified during fermentation: 15 alcohols, 18 ketones, 4 pyrazines, 4 esters, 10 acids, 5 phenols, 3 hydrocarbons, 3 amines, 2 aldehydes, 5 ethers, and 5 other components. The number and levels of VOCs differed depending on the fermentation duration. Most VOCs (62 compounds) were identified in the 48hFL. Inhibition rates of the VOCs all reached their peaks in the 24hFL. Significant (*P* < 0.05) positive correlations between levels of 2,4-di-*tert*-butylphenol, 1-octanol, and benzothiazole and the rates of *M. fructicola* and *C. gloeosporioides* inhibition were observed. Benzoic acid and benzaldehyde levels showed significant (*P* < 0.05) positive correlations with the rates of *M. fructicola* inhibition, and anisole and 3-methylbutanal showed the same against *C. gloeosporioides*. Treatment with 2,4-di-*tert*-butylphenol showed strong inhibitory effects on both *M. fructicola* and *C. gloeosporioides in vitro*. Benzothiazole showed the strongest inhibitory effects on the mycelial extension of both *M. fructicola* and *C. gloeosporioides in vivo*, and also increased the healthy fruit rate. Our results demonstrated that 2,4-di-*ter*t-butylthiophenol and benzothiazole are key inhibitory VOCs produced by *B. subtilis* CF-3. These results lay a preliminary foundation for the clarification of key biocontrol VOCs and provide a theoretical basis for improving biocontrol efficiency and application of *B. subtilis* strains in the biocontrol field.

## Author contributions

HG, PL, XX, and WG designed the experiments. PL and XX performed the experiments. PL, XX, and QZ analyzed the data. HG and PL drafted the manuscript. All authors read and approved the final manuscript.

### Conflict of interest statement

The authors declare that the research was conducted in the absence of any commercial or financial relationships that could be construed as a potential conflict of interest.

## References

[B1] Arrebola E. Sivakumar D. Korsten L. (2010). Effect of volatile compounds produced by *Bacillus* strains on postharvest decay in citrus. Biol. Control 53, 122–128. 10.1016/j.biocontrol.2009.11.010

[B2] Banani H. Spadaro D. Zhang D. Matic S. Garibaldi A. Guilin M. L. (2015). Postharvest application of a novel chitinase cloned from *Metschnikowia fructicola* and overexpressed in *Pichia pastoris* to control brown rot of peaches. Int. J. Food Microbiol. 199, 54–61. 10.1016/j.ijfoodmicro.2015.01.002 25632799

[B3] Belghit S. Driche E. H. Bijani C. Zitouni A. Sabaou N. Badji B.. (2016). Activity of 2,4-Di-*tert*-butylphenol produced by a strain of *Streptomyces mutabilis* isolated from a Saharan soil against *Candida albicans* and other pathogenic fungi. J. Mycol. Med. 26, 160–169. 10.1016/j.mycmed.2016.03.001 27107984

[B4] Casals C. Elmer P. A. G. Vinas I. Teixido N. Sisquella M. Usall J. (2012). The combination of curing with either chitosan or *Bacillus subtilis* CPA-8 to control brown rot infections caused by *Monilinia fructicola*. Postharvest Biol. Tec. 64, 126–132. 10.1016/j.postharvbio.2011.06.004

[B5] Chen H. Xiao X. Wang J. Wu L. Zheng Z. Yu Z. (2008). Antagonistic effects of volatiles generated by *Bacillus subtilis* on spore germination and hyphal growth of the plant pathogen *Botrytis cinerea*. Biotechnol. Lett. 30, 919–923. 10.1007/s10529-007-9626-9 18165869

[B6] Dharni S. Sanchita Maurya A. Samad A. Srivastava S. K. Sharma A.. (2014). Purification, characterization, and *in vitro* activity of 2,4-Di-*tert*-butylphenol from *Pseudomonas monteilii* PsF84: conformational and molecular docking studies. J. Agric. Food Chem. 62, 6138–6146. 10.1021/jf5001138 24934765

[B7] Fischer J. M. M. Savi D. C. Aluizio R. May De Mio L. L. Glienke C. (2017). Characterization of *Monilinia* species associated with brown rot in stone fruit in Brazil. Plant Pathol. 66, 423–436. 10.1111/ppa.12578

[B8] Gao H. Y. Xu X. X. Dai Y. W. He H. X. (2016). Isolation, identification and characterization of *Bacillus subtilis* CF-3, a bacterium from fermented bean curd for controlling postharvest diseases of peach fruit. Food Sci. Technol. Res. 22, 377–385. 10.3136/fstr.22.377

[B9] Gao H. Y. Xu X. X. Zeng Q. Li P. Z. (2017). Optimization of headspace Solid-phase microextraction for GC-MS analysis of volatile compounds produced by biocontrol strain *Bacillus subtilis* CF-3 using response surface methodology. Food Sci. Technol. Res. 23, 583–593. 10.3136/fstr.23.583

[B10] Gao Z. F. Zhang B. J. Liu H. P. Han J. C. Zhang Y. J. (2017). Identification of endophytic *Bacillus velezensis* ZSY-1 strain and antifungal activity of its volatile compounds against *Alternaria solani* and *Botrytis cinerea*. Biol. Control 105, 27–39. 10.1016/j.biocontrol.2016.11.007

[B11] Gillot G. Decourcelle N. Dauer G. Barbier G. Coton E. Delmail D.. (2016). 1-Octanol, a self-inhibitor of spore germination in *Penicillium camemberti*. Food Microbiol. 57, 1–7. 10.1016/j.fm.2015.12.008 27052695

[B12] Jiang C. M. Shi J. L. Liu Y. L. Zhu C. Y. (2014). Inhibition of *Aspergillus carbonarius* and fungal contamination in table grapes using *Bacillus subtilis*. Food Control 35, 41–48. 10.1016/j.foodcont.2013.06.054

[B13] Keri R. S. Patil M. R. Patil S. A. Budagumpi S. (2015). A comprehensive review in current developments of benzothiazole based molecules in medicinal chemistry. Eur. J. Med. Chem. 89, 207–251. 10.1016/j.ejmech.2014.10.059 25462241

[B14] Landum M. C. Felix M. D. Alho J. Garcia R. Cabrita M. J. Rei F.. (2016). Antagonistic activity of fungi of *Olea europaea* L. against *Colletotrichum acutatum*. Microbiol. Res. 183, 100–108. 10.1016/j.micres.2015.12.001 26805623

[B15] Leelasuphakul W. Hemmanee P. Chuenchitt S. (2008). Growth inhibitory properties of *Bacillus subtilis* strains and their metabolites against the green mold pathogen (*Penicillium digitatum* Sacc.) of citrus fruit. Postharvest Biol. Tec. 48, 113–121. 10.1016/j.postharvbio.2007.09.024

[B16] Liu Y. Tao J. Yan Y. J. Li B. Li H. Li C. (2011). Biocontrol efficiency of *Bacillus subtilis* SL-13 and characterization of an antifungal chitinase. Chinese J. Chem. Eng. 19, 128–134. 10.1016/S1004-9541(09)60188-9

[B17] Ma X. Wang X. B. Cheng J. Nie X. Yu X. X. Zhao Y. T. (2015). Microencapsulation of *Bacillus subtilis* B99-2 and its biocontrol efficiency against *Rhizoctonia solani* in tomato. Biol. Control 90, 34–41. 10.1016/j.biocontrol.2015.05.013

[B18] Maachia B. Rafik E. Chérif M. Nandal P. Mohapatra T. Bernard P. (2015). Biological control of the grapevine diseases ‘grey mold’ and ‘powdery mildew’ by *Bacillus* B27 and B29 strains. Indian J. Exp. Biol. 53, 109–115. 25757242

[B19] Mari M. Martini C. Guidarelli M. Neri F. (2012). Postharvest biocontrol of *Monilinia laxa, Monilinia fructicola* and *Monilinia fructigena* on stone fruit by two *Aureobasidium pullulans* strains. Biol. Control 60, 132–140. 10.1016/j.biocontrol.2011.10.013

[B20] Munir M. Amsden B. Dixon E. Vaillancourt L. Gauthier N. A. W. (2016). Characterization of *Colletotrichum* species causing bitter rot of apple in Kentucky Orchards. Plant Dis. 100, 2194–2203. 10.1094/PDIS-10-15-1144-RE 30682908

[B21] Ongena M. Jourdan E. Adam A. Paquot M. Brans A. Joris B.. (2007). Surfactin and fengycin lipopeptides of *Bacillus subtilis* as elicitors of induced systemic resistance in plants. Environ. Microbiol. 9, 1084–1090. 10.1111/j.1462-2920.2006.01202.x 17359279

[B22] Perez C. Agnese A. M. Cabrera J. L. (1999). The essential oil of *Senecio graveolens* (*Compositae*): chemical composition and antimicrobial activity tests. J. Ethnopharmacol. 66, 91–96. 10.1016/S0378-8741(98)00204-9 10432213

[B23] Prusky D. (2011). Reduction of the incidence of postharvest quality losses, and future prospects. Food Secur. 3, 463–474. 10.1007/s12571-011-0147-y

[B24] Schreinemachers P. Tipraqsa P. (2012). Agricultural pesticides and land use intensification in high, middle and low income countries. Food Policy 37, 616–626. 10.1016/j.foodpol.2012.06.003

[B25] Senthil R. Prabakar K. Rajendran L. Karthikeyan G. (2011). Efficacy of different biological control agents against major postharvest pathogens of grapes under room temperature storage conditions. Phytopathol. Mediterr. 50, 55–65.

[B26] Sivakumar D. Arrebola E. Korsten L. (2008). Postharvest decay control and quality retention in litchi (cv. McLean's Red) by combined application of modified atmosphere packaging and antimicrobial agents. Crop Prot. 27, 1208–1214. 10.1016/j.cropro.2008.03.002

[B27] Spadoni A. Guidarelli M. Phillips J. Mari M. Wisniewski M. (2015). Transcriptional profiling of apple fruit in response to heat treatment: involvement of a defense response during *Penicillium expansum* infection. Postharvest Biol. Technol. 101, 37–48. 10.1016/j.postharvbio.2014.10.009

[B28] Torres M. J. Brandan C. P. Petroselli G. Erra-Balsells R. Audisio M. C. (2016). Antagonistic effects of *Bacillus subtilis* subsp *subtilis* and *B*. amyloliquefaciens against Macrophomina phaseolina: SEM study of fungal changes and UV-MALDI-TOF MS analysis of their bioactive compounds. Microbiol. Res. 182, 31–39. 10.1016/j.micres.2015.09.005 26686611

[B29] Ugolini L. Martini C. Lazzeri L. D'Avino L. Mari M. (2014). Control of postharvest grey mould (*Botrytis cinerea* Per.: Fr.) on strawberries by glucosinolate-derived allyl-isothiocyanate treatments. Postharvest Biol. Technol. 90, 34–39. 10.1016/j.postharvbio.2013.12.002

[B30] Varsha K. K. Devendra L. Shilpa G. Priya S. Pandey A. Nampoothiri K. M. (2015). 2,4-Di-*tert*-butyl phenol as the antifungal, antioxidant bioactive purified from a newly isolated *Lactococcus* sp. Int. J. Food Microbiol. 211, 44–50. 10.1016/j.ijfoodmicro.2015.06.025 26164257

[B31] Wang C. J. Liu Z. Q. (2007). Foliar uptake of pesticides - present status and future challenge. Pestic. Biochem. Physiol. 87, 1–8. 10.1016/j.pestbp.2006.04.004

[B32] Wang J. Xia X. M. Wang H. Y. Li P. P. Wang K. Y. (2013). Inhibitory effect of lactoferrin against gray mould on tomato plants caused by *Botrytis cinerea* and possible mechanisms of action. Int. J. Food Microbiol. 161, 151–157. 10.1016/j.ijfoodmicro.2012.11.025 23333340

[B33] Wang S. L. Yeh P. Y. (2008). Purification and characterization of a chitosanase from a nattokinase producing strain *Bacillus subtilis* TKU007. Process Biochem. 43, 132–138. 10.1016/j.procbio.2007.11.002

[B34] Wang Y. Li Y. X. Yang J. L. Ruan J. Sun C. J. (2016). Microbial volatile organic compounds and their application in microorganism identification in foodstuff. Trac-Trends Anal. Chem. 78, 1–16. 10.1016/j.trac.2015.08.010

[B35] Xu C. Wang C. S. Ju L. L. Zhang R. Biggs A. R. Tanaka E. (2015). Multiple locus genealogies and phenotypic characters reappraise the causal agents of apple ring rot in China. Fungal Divers. 71, 215–231. 10.1007/s13225-014-0306-5

[B36] Yan J. Q. Yuan S. Z. Wang C. Y. Ding X. Y. Cao J. K. Jiang W. B. (2015). Enhanced resistance of jujube (*Zizyphus jujuba Mill*. cv. Dongzao) fruit against postharvest Alternaria rot by beta-aminobutyric acid dipping. Sci. Hortic. 186, 108–114. 10.1016/j.scienta.2015.02.018

[B37] Zhang Y. Y. Zeng L. Z. Yang J. L. Zheng X. D. Yu T. (2015). 6-Benzylaminopurine inhibits growth of *Monilinia fructicola* and induces defense-related mechanism in peach fruit. Food Chem. 187, 210–217. 10.1016/j.foodchem.2015.04.100 25977018

[B38] Zheng M. Shi J. Y. Shi J. Wang Q. G. Li Y. H. (2013). Antimicrobial effects of volatiles produced by two antagonistic *Bacillus* strains on the anthracnose pathogen in postharvest mangos. Biol. Control 65, 200–206. 10.1016/j.biocontrol.2013.02.004

